# Supplementary material for: Temperature-Dependent Effects of Induced Hyperthermia, Including Whole-Body Hyperthermia, on the Hallmarks of Cancer: A Systematic Review
Source: Cancers (Basel). 2025 Nov 28;17(23):3824. doi: 10.3390/cancers17233824 (PMC12691179; doi:10.3390/cancers17233824)
Supplement: Supplementary file 1 [file cancers-17-03824-s001.zip › cancers-3995488-supplementary/Supplementary File S2 Hyperthermia_Hallmarks of Cancer_Summary_Table.pdf]

Hallmark of Cancer	Main findings from reviewed literature	Temperature range	References
Avoiding immune destruction	Mild HT enhances antitumor immunity by increasing NK and CTL cytotoxicity, improving APC maturation, and boosting lymphocyte survival. HSP70/90 act as DAMPs, increasing antigen presentation. Cytokine release enhances immune recruitment. High or repeated HT suppresses immunity through immunecell apoptosis and anti-inflammatory cytokines.	39–41°C: immune activation; >42°C: immune suppression, thermotolerance	[1–14]
Genome Instability & Mutation	HT destabilizes DNA replication and repair by inhibiting polymerases α/β, stalling replication forks, and inducing SSBs/DSBs. Nuclear matrix disruption impairs BER and other pathways. Homologous recombination proteins are destabilized leading to genomic instability.	>41°C: DNA destabilization; peak effects at 41–43°C	[6,12,13,15–30]
Resisting cell death	Apoptosis dominates at moderate temperatures; HSP70 may rescue apoptotic signaling in p53-mutant cells. Necrosis appears at higher ranges and is amplified with radiotherapy in even lower temperatures. Autophagy promotes protein degradation and damage control.	41–43°C: apoptosis; >41.5°C: necrosis; 40–42°C: autophagy	[1,2,6,9,12,13,15,17,21,22,31–43]
Deregulating cellular energetics	HT suppresses mitochondrial ATP production and induces glycolytic and oxidative stress. ROS generation increases via mitochondrial and enzymatic sources, damaging DNA and activating apoptosis or autophagy. Inhibition of pentose Phosphate pathway reduces antioxidant capacity.	>41°C: metabolic suppression; 39–41°C: mild metabolic stress	[1,14,33,40,44–54]

Inducing angiogenesis	Mild HT increases perfusion and oxygenation. Higher temperatures cause vascular collapse through coagulation, thrombosis, and RBC deformation. VEGF vs. PAI-1 balance under HIF-1 determines Angiogenic response.	<42°C: improved perfusion; 42°C: vascular collapse	[13,14,17,18,44,48,54–60]
Activating invasion and metastasis	HT may promote metastasis through HSF-1 activation, EMT pathways, and MTA-1 upregulation.HT disruptsmembrane integrity and Cytoskeletal stability, increasing permeability, drug uptake, and immune recognition.	>42°C: prometastatic signaling;	[1,2,12,13,17,18,22,23,25,31,41,53,56,61–64]
Enabling replicative immortality	HT stabilizes telomerase via HSP23, 70, 90, preventing degradation. Telomerase activity may increase due to structural stabilization.	39–41°C: telomerase stabilization; >42°C: potential destabilization	[65–68]

References

1. Frey, B.; Weiss, E.-M.; Rubner, Y.; Wunderlich, R.; Ott, O.J.; Sauer, R.; Fietkau, R.; Gaipl, U.S. Old and New Facts about Hyperthermia-Induced Modulations of the Immune System. *Int. J. Hyperth.* **2012**, *28*, 528–542. https://doi.org/10.3109/02656736.2012.677933.
2. Dieing, A.; Ahlers, O.; Hildebrandt, B.; Kerner, T.; Tamm, I.; Possinger, K.; Wust, P. The Effect of Induced Hyperthermia on the Immune System. *Prog. Brain Res.* **2007**, *162*, 137–152. https://doi.org/10.1016/S0079-6123(06)62008-6. Available online: http://sfxit.ugent.be/ugent?sid=EMBASE&issn=00796123&id=doi:10.1016%2FS0079-6123%2806%2962008-6&atitle=The+effect+of+induced+hyperthermia+on+the+immune+system&stitle=Prog.+Brain+Res.&title=Progress+in+Brain+Research&volume=162&issue=&spage=137&epage=152&aulast=Dieing&aufirst=Annette&auinit=A.&aufull=Dieing+A.&coden=PBRRA&isbn=volume9780444519269&pages=137-152&date=2007&auinit1=A&auinitm= (accessed on 26 November 2025).
3. Zhang, H.-G.; Mehta, K.; Cohen, P.; Guha, C. Hyperthermia on Immune Regulation: A Temperature's Story. *Cancer Lett.* **2008**, *271*, 191–204. https://doi.org/10.1016/j.canlet.2008.05.026.
4. Baronzio, G.; Gramaglia, A.; Fiorentini, G. Hyperthermia and Immunity. *A Brief. Overview. Vivo* **2006**, *20*, 689–695.
5. Van Dieren, L.; Quisenaerts, T.; Licata, M.; Beddok, A.; Lellouch, A.G.; Ysebaert, D.; Saldien, V.; Peeters, M.; Gorbaslieva, I. Combined Radiotherapy and Hyperthermia: A Systematic Review of Immunological Synergies for Amplifying Radiation-Induced Abscopal Effects. *Cancers* **2024**, *16*, 3656.
6. Lee, S.; Son, B.; Park, G.; Kim, H.; Kang, H.; Jeon, J.; Youn, H.; Youn, B. Immunogenic Effect of Hyperthermia on Enhancing Radiotherapeutic Efficacy. *Int. J. Mol. Sci.* **2018**, *19*, 2795. https://doi.org/10.3390/ijms19092795.

7. Ito, A.; Honda, H.; Kobayashi, T. Cancer Immunotherapy Based on Intracellular Hyperthermia Using Magnetite Nanoparticles: A Novel Concept of "Heat-Controlled Necrosis" with Heat Shock Protein Expression. *Cancer Immunol. Immunother.* **2006**, *55*, 320–328. https://doi.org/10.1007/s00262-005-0049-y.
8. Manjili, M.H.; Wang, X.Y.; Park, J.; Macdonald, I.J.; Li, Y.; Van Schie, R.C.A.A.; Subjeck, J.R. Cancer Immunotherapy: Stress Proteins and Hyperthermia. *Int. J. Hyperth.* **2002**, *18*, 506–520. https://doi.org/10.1080/02656730110116696.
9. Skitzki, J.J.; Repasky, E.A.; Evans, S.S. Hyperthermia as an Immunotherapy Strategy for Cancer. *Curr. Opin. Investig. Drugs* **2009**, *10*, 550–558.
10. Appenheimer, M.M.; Evans, S.S. Temperature and Adaptive Immunity. *Handb. Clin. Neurol.* **2018**, *156*, 397–415. https://doi.org/10.1016/B978-0-444-63912-7.00024-2.
11. Muthana, M.; Multhoff, G.; Pockley, A.G. Tumour Infiltrating Host Cells and Their Significance for Hyperthermia. *Int. J. Hyperth.* **2010**, *26*, 247–255. https://doi.org/10.3109/02656730903413375.
12. Toraya-Brown, S.; Fiering, S. Local Tumour Hyperthermia as Immunotherapy for Metastatic Cancer. *Int. J. Hyperth.* **2014**, *30*, 531–539. https://doi.org/10.3109/02656736.2014.968640.
13. Pennacchioli, E.; Fiore, M.; Gronchi, A. Hyperthermia as an Adjunctive Treatment for Soft-Tissue Sarcoma. *Expert. Rev. Anticancer Ther.* **2009**, *9*, 199–210. https://doi.org/10.1586/14737140.9.2.199.
14. Dewhirst, M.W.; Lee, C.-T.; Ashcraft, K.A. The Future of Biology in Driving the Field of Hyperthermia. *Int. J. Hyperth.* **2016**, *32*, 4–13. https://doi.org/10.3109/02656736.2015.1091093.
15. Hader, M.; Frey, B.; Fietkau, R.; Hecht, M.; Gaipl, U.S. Immune Biological Rationales for the Design of Combined Radio- and Immunotherapies. *Cancer Immunol. Immunother.* **2020**, *69*, 293–306. https://doi.org/10.1007/s00262-019-02460-3.
16. Lauber, K.; Brix, N.; Ernst, A.; Hennel, R.; Krombach, J.; Anders, H.; Belka, C. Targeting the Heat Shock Response in Combination with Radiotherapy: Sensitizing Cancer Cells to Irradiation-Induced Cell Death and Heating up Their Immunogenicity. *Cancer Lett.* **2015**, *368*, 209–229.
17. Hildebrandt, B.; Wust, P.; Ahlers, O.; Dieing, A.; Sreenivasa, G.; Kerner, T.; Felix, R.; Riess, H. The Cellular and Molecular Basis of Hyperthermia. *Crit. Rev. Oncol. Hematol.* **2002**, *43*, 33–56.
18. Gao, S.; Zheng, M.; Ren, X.; Tang, Y.; Liang, X. Local Hyperthermia in Head and Neck Cancer: Mechanism, Application and Advance. *Oncotarget* **2016**, *7*, 57367–57378. https://doi.org/10.18632/oncotarget.10350.
19. Dewhirst, M.W.; Lora-Michiels, M.; Viglianti, B.L.; Dewey, W.C.; Repacholi, M. Carcinogenic Effects of Hyperthermia. *Int. J. Hyperth.* **2003**, *19*, 236–251. https://doi.org/10.1080/0265673031000070811.
20. Eppink, B.; Krawczyk, P.M.; Stap, J.; Kanaar, R. Hyperthermia-Induced DNA Repair Deficiency Suggests Novel Therapeutic Anti-Cancer Strategies. *Int. J. Hyperth.* **2012**, *28*, 509–517. https://doi.org/10.3109/02656736.2012.695427.
21. Turner, T.; Caspari, T. When Heat Casts a Spell on the DNA Damage Checkpoints. *Open Biol.* **2014**, *4*, 140008. https://doi.org/10.1098/rsob.140008.
22. Youssef, I.; Zulfiqar, H.; Amin, N.P. Hyperthermia for Chest Wall Recurrence. In *StatPearls*; StatPearls Publishing: Treasure Island, FL, USA, 2020.
23. Roti Roti, J.L. Cellular Responses to Hyperthermia (40–46 Degrees C): Cell Killing and Molecular Events. *Int. J. Hyperth.* **2008**, *24*, 3–15. https://doi.org/10.1080/02656730701769841.
24. Ohnishi, T. The Role of the P53 Molecule in Cancer Therapies with Radiation and/or Hyperthermia. *J. Cancer Res. Ther.* **2005**, *1*, 147–150. https://doi.org/10.4103/0973-1482.19594.

25. Ahmed, K.; Tabuchi, Y.; Kondo, T. Hyperthermia: An Effective Strategy to Induce Apoptosis in Cancer Cells. *Apoptosis* **2015**, *20*, 1411–1419. https://doi.org/10.1007/s10495-015-1168-3.
26. Walther, W.; Stein, U. Heat-Responsive Gene Expression for Gene Therapy. *Adv. Drug Deliv. Rev.* **2009**, *61*, 641–649. https://doi.org/10.1016/j.addr.2009.02.009.
27. Mantso, T.; Goussetis, G.; Franco, R.; Botaitis, S.; Pappa, A.; Panayiotidis, M. Effects of Hyperthermia as a Mitigation Strategy in DNA Damage-Based Cancer Therapies. *Semin. Cancer Biol.* **2016**, *37–38*, 96–105. https://doi.org/10.1016/j.semcancer.2016.03.004.
28. van den Tempel, N.; Horsman, M.R.; Kanaar, R. Improving Efficacy of Hyperthermia in Oncology by Exploiting Biological Mechanisms. *Int. J. Hyperth.* **2016**, *32*, 446–454. https://doi.org/10.3109/02656736.2016.1157216.
29. Iliakis, G.; Wu, W.; Wang, M. DNA Double Strand Break Repair Inhibition as a Cause of Heat Radiosensitization: Re-Evaluation Considering Backup Pathways of NHEJ. *Int. J. Hyperth.* **2008**, *24*, 17–29. https://doi.org/10.1080/02656730701784782.
30. Issels, R.; Kampmann, E.; Kanaar, R.; Lindner, L.H. Hallmarks of Hyperthermia in Driving the Future of Clinical Hyperthermia as Targeted Therapy: Translation into Clinical Application. *Int. J. Hyperth.* **2016**, *32*, 89–95. https://doi.org/10.3109/02656736.2015.1119317.
31. Ahmed, K.; Zaidi, S.F. Treating Cancer with Heat: Hyperthermia as Promising Strategy to Enhance Apoptosis. *J. Pak. Med. Assoc.* **2013**, *63*, 504–508.
32. Jolly, C.; Morimoto, R.I. Role of the Heat Shock Response and Molecular Chaperones in Oncogenesis and Cell Death. *J. Natl. Cancer Inst.* **2000**, *92*, 1564–1572. https://doi.org/10.1093/jnci/92.19.1564.
33. Ahmed, K.; Zaidi, S.F.; Rehman, M.U.; Rehman, R.; Kondo, T. Hyperthermia and Protein Homeostasis: Cytoprotection and Cell Death. *J. Therm. Biol.* **2020**, *91*, 102615. https://doi.org/10.1016/j.jtherbio.2020.102615.
34. Roufayel, R.; Kadry, S. Expression of MiR-23a by Apoptotic Regulators in Human Cancer: A Review. *Cancer Biol. Ther.* **2017**, *18*, 269–276. https://doi.org/10.1080/15384047.2017.1310342.
35. Kejík, Z.; Jakubek, M.; Kaplánek, R.; Králová, J.; Mikula, I.; Martásek, P.; Král, V. Epigenetic Agents in Combined Anticancer Therapy. *Future Med. Chem.* **2018**, *10*, 1113–1130. https://doi.org/10.4155/fmc-2017-0203.
36. Milleron, R.S.; Bratton, S.B. "Heated" Debates in Apoptosis. *Cell Mol. Life Sci.* **2007**, *64*, 2329–2333. https://doi.org/10.1007/s00018-007-7135-6.
37. Bai, J.F.; Liu, P.; Xu, L.X. Recent Advances in Thermal Treatment Techniques and Thermally Induced Immune Responses against Cancer. *IEEE Trans. Biomed. Eng.* **2014**, *61*, 1497–1505. https://doi.org/10.1109/tbme.2014.2314357.
38. Frey, B.; Ruckert, M.; Deloch, L.; Ruhle, P.F.; Derer, A.; Fietkau, R.; Gaipl, U.S. Immunomodulation by Ionizing Radiation-Impact for Design of Radio-Immunotherapies and for Treatment of Inflammatory Diseases. *Immunol. Rev.* **2017**, *280*, 231–248. https://doi.org/10.1111/imr.12572.
39. Weiss, E.-M.; Frey, B.; Rodel, F.; Herrmann, M.; Schlucker, E.; Voll, R.E.; Fietkau, R.; Gaipl, U.S. Ex Vivo- and in Vivo-Induced Dead Tumor Cells as Modulators of Antitumor Responses. *Ann. N. Y. Acad. Sci.* **2010**, *1209*, 109–117. https://doi.org/10.1111/j.1749-6632.2010.05743.x.
40. Schildkopf, P.; Ott, O.J.; Frey, B.; Wadepohl, M.; Sauer, R.; Fietkau, R.; Gaipl, U.S. Biological Rationales and Clinical Applications of Temperature Controlled Hyperthermia–Implications for Multimodal Cancer Treatments. *Curr. Med. Chem.* **2010**, *17*, 3045–3057. https://doi.org/10.2174/092986710791959774.
41. Calderwood, S.K.; Ciocca, D.R. Heat Shock Proteins: Stress Proteins with Janus-like Properties in Cancer. *Int. J. Hyperth.* **2008**, *24*, 31–39. https://doi.org/10.1080/02656730701858305.

42. Zhang, Y.; Calderwood, S.K. Autophagy, Protein Aggregation and Hyperthermia: A Mini-Review. *Int. J. Hyperth.* **2011**, *27*, 409–414. https://doi.org/10.3109/02656736.2011.552087.
43. Xie, W.-Y.; Zhou, X.-D.; Yang, J.; Chen, L.-X.; Ran, D.-H. Inhibition of Autophagy Enhances Heat-Induced Apoptosis in Human Non-Small Cell Lung Cancer Cells through ER Stress Pathways. *Arch. Biochem. Biophys.* **2016**, *607*, 55–66. https://doi.org/10.1016/j.abb.2016.08.016.
44. Kroemer, G.; Pouyssegur, J. Tumor Cell Metabolism: Cancer's Achilles' Heel. *Cancer Cell* **2008**, *13*, 472–482.
45. Kennedy, K.M.; Dewhirst, M.W. Tumor Metabolism of Lactate: The Influence and Therapeutic Potential for MCT and CD147 Regulation. *Futur. Oncol*. **2009**, *6*, 127–148.
46. Abdelnour, S.A.; Abd El-Hack, M.E.; Khafaga, A.F.; Arif, M.; Taha, A.E.; Noreldin, A.E. Stress Biomarkers and Proteomics Alteration to Thermal Stress in Ruminants: A Review. *J. Therm. Biol.* **2019**, *79*, 120–134. https://doi.org/10.1016/j.jtherbio.2018.12.013.
47. Vaupel, P.W.; Kelleher, D.K. Blood Flow and Associated Pathophysiology of Uterine Cervix Cancers: Characterisation and Relevance for Localised Hyperthermia. *Int. J. Hyperth.* **2012**, *28*, 518–527. https://doi.org/10.3109/02656736.2012.699134.
48. Vaupel, P.W.; Kelleher, D.K. Pathophysiological and Vascular Characteristics of Tumours and Their Importance for Hyperthermia: Heterogeneity Is the Key Issue. *Int. J. Hyperth.* **2010**, *26*, 211–223. https://doi.org/10.3109/02656731003596259.
49. Yi, G.Y.; Kim, M.J.; Kim, H.I.; Park, J.; Baek, S.H. Hyperthermia Treatment as a Promising Anti-Cancer Strategy: Therapeutic Targets, Perspective Mechanisms and Synergistic Combinations in Experimental Approaches. *Antioxidants* **2022**, *11*, 625.
50. Liso, A.; Capitanio, N.; Gerli, R.; Conese, M. From Fever to Immunity: A New Role for IGFBP-6? *J. Cell Mol. Med.* **2018**, *22*, 4588–4596. https://doi.org/10.1111/jcmm.13738.
51. Gius, D.; Mattson, D.; Bradbury, C.M.; Smart, D.K.; Spitz, D.R. Thermal Stress and the Disruption of Redox-Sensitive Signalling and Transcription Factor Activation: Possible Role in Radiosensitization. *Int. J. Hyperth.* **2004**, *20*, 213–223. https://doi.org/10.1080/02656730310001619505.
52. Coss, R.A. Inhibiting Induction of Heat Shock Proteins as a Strategy to Enhance Cancer Therapy. *Int. J. Hyperth.* **2005**, *21*, 695–701. https://doi.org/10.1080/02656730500331918.
53. Wust, P.; Hildebrandt, B.; Sreenivasa, G.; Rau, B.; Gellermann, J.; Riess, H.; Felix, R.; Schlag, P.M. Hyperthermia in Combined Treatment of Cancer. *Lancet Oncol.* **2002**, *3*, 487–497. https://doi.org/10.1016/s1470-2045(02)00818-5.
54. Hokland, S.L.; Nielsen, T.; Busk, M.; Horsman, M.R. Imaging Tumour Physiology and Vasculature to Predict and Assess Response to Heat. *Int. J. Hyperth.* **2010**, *26*, 264–272. https://doi.org/10.3109/02656730903585982.
55. Payne, M.; Bossmann, S.H.; Basel, M.T. Direct Treatment versus Indirect: Thermo-Ablative and Mild Hyperthermia Effects. *Wiley Interdiscip. Rev. Nanomed. Nanobiotech.* **2020**, *12*, e1638. https://doi.org/10.1002/wnan.1638.
56. Repasky, E.A.; Evans, S.S.; Dewhirst, M.W. Temperature Matters! And Why It Should Matter to Tumor Immunologists. *Cancer Immunol. Res.* **2013**, *1*, 210–216. https://doi.org/10.1158/2326-6066.CIR-13-0118.
57. Jones, R.G.; Thompson, C.B. Tumor Suppressors and Cell Metabolism: A Recipe for Cancer Growth. *Genes. Dev.* **2009**, *23*, 537–548.
58. Horsman, M.R. Angiogenesis and Vascular Targeting: Relevance for Hyperthermia. *Int. J. Hyperth.* **2008**, *24*, 57–65. https://doi.org/10.1080/02656730701829710.
59. Griffin, R.J.; Dings, R.P.M.; Jamshidi-Parsian, A.; Song, C.W. Mild Temperature Hyperthermia and Radiation Therapy: Role of Tumour Vascular Thermotolerance and Relevant Physiological Factors. *Int. J. Hyperth.* **2010**, *26*, 256–263. https://doi.org/10.3109/02656730903453546.
60. Horsman, M.R. Tissue Physiology and the Response to Heat. *Int. J. Hyperth.* **2006**, *22*, 197–203. https://doi.org/10.1080/02656730600689066.

61. Calderwood, S.K. Heat Shock Proteins in Breast Cancer Progression–a Suitable Case for Treatment? *Int. J. Hyperth.* **2010**, *26*, 681–685. https://doi.org/10.3109/02656736.2010.490254.
62. Lee, C.-T.; Mace, T.; Repasky, E.A. Hypoxia-Driven Immunosuppression: A New Reason to Use Thermal Therapy in the Treatment of Cancer? *Int. J. Hyperth.* **2010**, *26*, 232–246. https://doi.org/10.3109/02656731003601745.
63. Mahmood, J.; Shukla, H.D.; Soman, S.; Samanta, S.; Singh, P.; Kamlapurkar, S.; Saeed, A.; Amin, N.P.; Vujaskovic, Z. Immunotherapy, Radiotherapy, and Hyperthermia: A Combined Therapeutic Approach in Pancreatic Cancer Treatment. *Cancers* **2018**, *10*, 469. https://doi.org/10.3390/cancers10120469.
64. Csoboz, B.; Balogh, G.E.; Kusz, E.; Gombos, I.; Peter, M.; Crul, T.; Gungor, B.; Haracska, L.; Bogdanovics, G.; Torok, Z.; et al. Membrane Fluidity Matters: Hyperthermia from the Aspects of Lipids and Membranes. *Int. J. Hyperth.* **2013**, *29*, 491–499. https://doi.org/10.3109/02656736.2013.808765.
65. Pandita, T.K. Role of HSPs and Telomerase in Radiotherapy. *Int. J. Hyperth.* **2005**, *21*, 689–694. https://doi.org/10.1080/02656730500271700.
66. Ince, T.A.; Richardson, A.L.; Bell, G.W.; Saitoh, M.; Godar, S.; Karnoub, A.E.; Iglehart, J.D.; Weinberg, R.A. Transformation of Different Human Breast Epithelial Cell Types Leads to Distinct Tumor Phenotypes. *Cancer Cell* **2007**, *12*, 160–170. https://doi.org/10.1016/j.ccr.2007.06.013.
67. Zhang, H.; Herbert, B.S.; Pan, K.H.; Shay, J.W.; Cohen, S.N. Disparate Effects of Telomere Attrition on Gene Expression during Replicative Senescence of Human Mammary Epithelial Cells Cultured under Different Conditions. *Oncogene* **2004**, *23*, 6193–6198. https://doi.org/10.1038/sj.onc.1207834.
68. Artandi, S.E.; DePinho, R.A. Telomeres and Telomerase in Cancer. *Carcinogenesis* **2009**, *31*, 9–18.
